# Supplementary material for: A Theoretical Study of the Occupied and Unoccupied Electronic Structure of High- and Intermediate-Spin Transition Metal Phthalocyaninato (Pc) Complexes: VPc, CrPc, MnPc, and FePc
Source: Nanomaterials (Basel). 2020 Dec 28;11(1):54. doi: 10.3390/nano11010054 (PMC7824030; doi:10.3390/nano11010054)
Supplement: Supplementary file 1 [file nanomaterials-11-00054-s001.zip › Supplementary Material/Figure S1 - S2.docx]

A theoretical study of the occupied and unoccupied electronic structure of high- and intermediate-spin transition metal phthalocyaninato (Pc) complexes: VPc, CrPc, MnPc, and FePc.

Silvia Carlotto,*^a^ Mauro Sambi, ^a^ Francesco Sedona, ^a^ Andrea Vittadini^b^ and Maurizio Casarin*^a,b^

Supplementary Material

**Figure S1** 3D plot of the MnPc 6e_g_^↓^ MO. Displayed isosurfaces correspond to ±0.015 e^1/^ × A^-3/2^


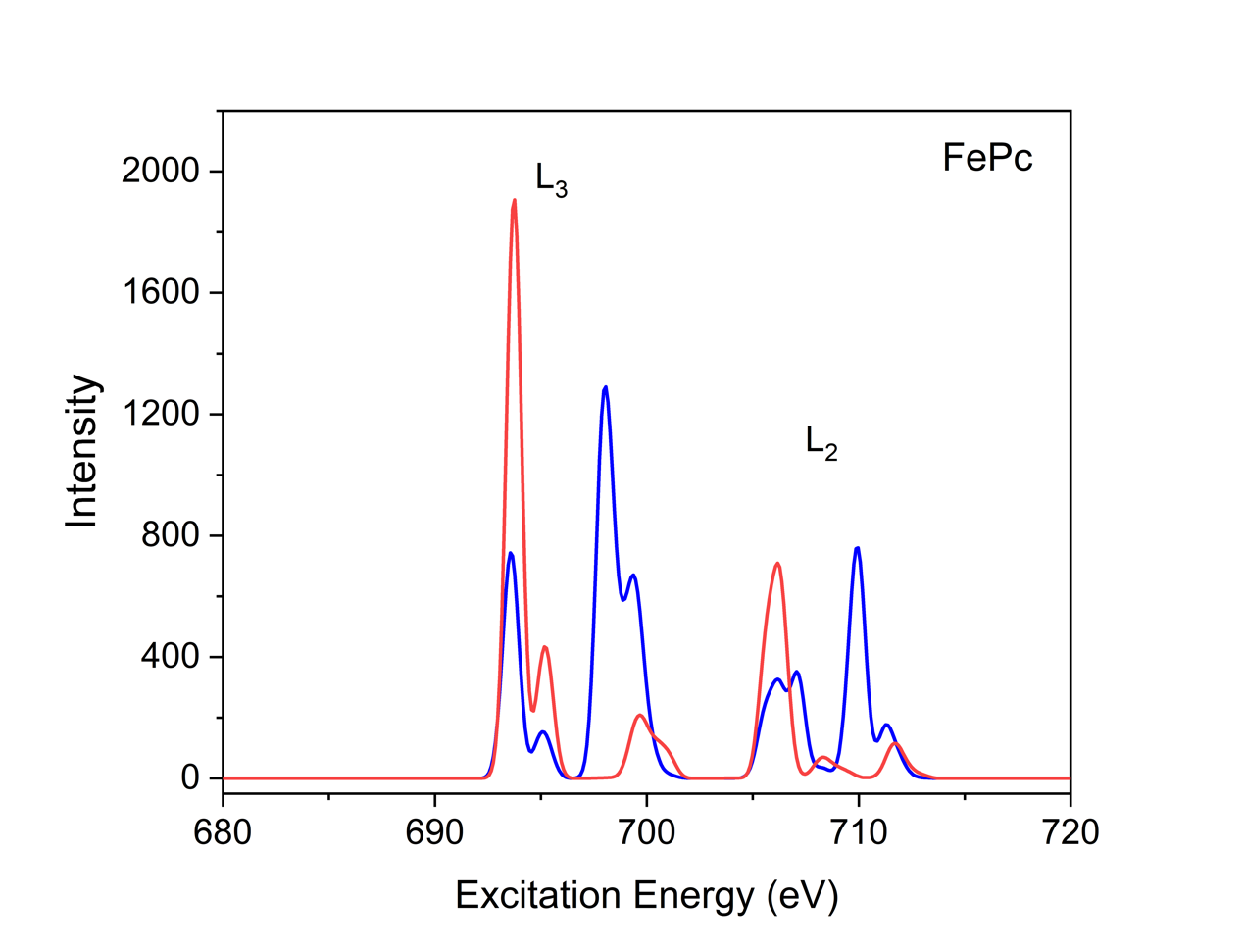


**Figure S2** ^IS^FePc ^||/⊥^*f*(*EE*) distributions estimated by adopting c_1_ = 0.18, c_2_ = 0.20, and c_3_ = 0.40. Blue and red lines correspond to || and ⊥ components, respectively. Simulated spectra have a Gaussian broadening of 1.5 eV**.**
