# Supplementary material for: A Theoretical Study of the Occupied and Unoccupied Electronic Structure of High- and Intermediate-Spin Transition Metal Phthalocyaninato (Pc) Complexes: VPc, CrPc, MnPc, and FePc
Source: Nanomaterials (Basel). 2020 Dec 28;11(1):54. doi: 10.3390/nano11010054 (PMC7824030; doi:10.3390/nano11010054)
Supplement: Supplementary file 1 [file nanomaterials-11-00054-s001.zip › Supplementary Material/Table S5.pdf]

Table S5 Symmetries of the $2p^{5}3d^{4/6}$ system.

	L	S	L+S	|L-S|	2(L+S)+1	2(L-S)+1	J=15/2	J=13/2	J=11/2	J=9/2	J=7/2	J=5/2	J=3/2	J=1/2	States	Deg.
^{6}F	3	2,5	5,5	0,5	12	2			$^{6}F_{11/2}$	$^{6}F_{9/2}$	$^{6}F_{7/2}$	$^{6}F_{5/2}$	$^{6}F_{3/2}$	$^{6}F_{1/2}$	6	42
^{6}D	2	2,5	4,5	0,5	10	2				$^{6}D_{9/2}$	$^{6}D_{7/2}$	$^{6}D_{5/2}$	$^{6}D_{3/2}$	$^{6}D_{1/2}$	5	30
^{6}P	1	2,5	3,5	1,5	8	4					$^{6}P_{7/2}$	$^{6}P_{5/2}$	$^{6}P_{3/2}$		3	18
^{4}F	3	1,5	4,5	1,5	10	4				$^{4}F_{9/2}$	$^{4}F_{7/2}$	$^{4}F_{5/2}$	$^{4}F_{3/2}$		4	28
^{4}D	2	1,5	3,5	0,5	8	2					$^{4}D_{7/2}$	$^{4}D_{5/2}$	$^{4}D_{3/2}$	$^{4}D_{1/2}$	4	20
^{4}P	1	1,5	2,5	0,5	6	2						$^{4}P_{5/2}$	$^{4}P_{3/2}$	$^{4}P_{1/2}$	3	12
^{4}I	6	1,5	7,5	4,5	16	10	$^{4}I_{15/2}$	$^{4}I_{13/2}$	$^{4}I_{11/2}$	$^{4}I_{9/2}$					4	52
^{4}H	5	1,5	6,5	3,5	14	8		$^{4}H_{13/2}$	$^{4}H_{11/2}$	$^{4}H_{9/2}$	$^{4}H_{7/2}$				4	44
^{4}G	4	1,5	5,5	2,5	12	6			$^{4}G_{11/2}$	$^{4}G_{9/2}$	$^{4}G_{7/2}$	$^{4}G_{5/2}$			4	36
^{2}I	6	0,5	6,5	5,5	14	12		$^{2}I_{13/2}$	$^{2}I_{11/2}$						2	26
^{2}H	5	0,5	5,5	4,5	12	10			$^{2}H_{11/2}$	$^{2}H_{9/2}$					2	22
^{2}G	4	0,5	4,5	3,5	10	8				$^{2}G_{9/2}$	$^{2}G_{7/2}$				2	18
^{4}H	5	1,5	6,5	3,5	14	8		$^{4}H_{13/2}$	$^{4}H_{11/2}$	$^{4}H_{9/2}$	$^{4}H_{7/2}$				4	44
^{4}G	4	1,5	5,5	2,5	12	6			$^{4}G_{11/2}$	$^{4}G_{9/2}$	$^{4}G_{7/2}$	$^{4}G_{5/2}$			4	36
^{4}F	3	1,5	4,5	1,5	10	4				$^{4}F_{9/2}$	$^{4}F_{7/2}$	$^{4}F_{5/2}$	$^{4}F_{3/2}$		4	28
^{2}H	5	0,5	5,5	4,5	12	10			$^{2}H_{11/2}$	$^{2}H_{9/2}$					2	22
^{2}G	4	0,5	4,5	3,5	10	8				$^{2}G_{9/2}$	$^{2}G_{7/2}$				2	18
^{2}F	3	0,5	3,5	2,5	8	6					$^{2}F_{7/2}$	$^{2}F_{5/2}$			2	14
^{4}G	4	1,5	5,5	2,5	12	6			$^{4}G_{11/2}$	$^{4}G_{9/2}$	$^{4}G_{7/2}$	$^{4}G_{5/2}$			4	36
^{4}F	3	1,5	4,5	1,5	10	4				$^{4}F_{9/2}$	$^{4}F_{7/2}$	$^{4}F_{5/2}$	$^{4}F_{3/2}$		4	28
^{4}D	2	1,5	3,5	0,5	8	2					$^{4}D_{7/2}$	$^{4}D_{5/2}$	$^{4}D_{3/2}$	$^{4}D_{1/2}$	4	20

^2G	4	0,5	4,5	3,5	10	8		$^2G_{9/2}$	$^2G_{7/2}$				2	18
^2F	3	0,5	3,5	2,5	8	6			$^2F_{7/2}$	$^2F_{5/2}$			2	14
^2D	2	0,5	2,5	1,5	6	4				$^2D_{5/2}$	$^2D_{3/2}$		2	10
^4G	4	1,5	5,5	2,5	12	6	$^4G_{11/2}$	$^4G_{9/2}$	$^4G_{7/2}$	$^4G_{5/2}$			4	36
^4F	3	1,5	4,5	1,5	10	4		$^4F_{9/2}$	$^4F_{7/2}$	$^4F_{5/2}$	$^4F_{3/2}$		4	28
^4D	2	1,5	3,5	0,5	8	2			$^4D_{7/2}$	$^4D_{5/2}$	$^4D_{3/2}$	$^4D_{1/2}$	4	20
^2G	4	0,5	4,5	3,5	10	8		$^2G_{9/2}$	$^2G_{7/2}$				2	18
^2F	3	0,5	3,5	2,5	8	6			$^2F_{7/2}$	$^2F_{5/2}$			2	14
^2D	2	0,5	2,5	1,5	6	4				$^2D_{5/2}$	$^2D_{3/2}$		2	10
^4F	3	1,5	4,5	1,5	10	4		$^4F_{9/2}$	$^4F_{7/2}$	$^4F_{5/2}$	$^4F_{3/2}$		4	28
^4D	2	1,5	3,5	0,5	8	2			$^4D_{7/2}$	$^4D_{5/2}$	$^4D_{3/2}$	$^4D_{1/2}$	4	20
^4P	1	1,5	2,5	0,5	6	2				$^4P_{5/2}$	$^4P_{3/2}$	$^4P_{1/2}$	3	12
^2F	3	0,5	3,5	2,5	8	6			$^2F_{7/2}$	$^2F_{5/2}$			2	14
^2D	2	0,5	2,5	1,5	6	4				$^2D_{5/2}$	$^2D_{3/2}$		2	10
^2P	1	0,5	1,5	0,5	4	2					$^2P_{3/2}$	$^2P_{1/2}$	2	6
^4D	2	1,5	3,5	0,5	8	2			$^4D_{7/2}$	$^4D_{5/2}$	$^4D_{3/2}$	$^4D_{1/2}$	4	20
^4P	1	1,5	2,5	0,5	6	2				$^4P_{5/2}$	$^4P_{3/2}$	$^4P_{1/2}$	3	12
^4S	0	1,5	1,5	1,5	4	4					$^4S_{3/2}$		1	4
^2D	2	0,5	2,5	1,5	6	4				$^2D_{5/2}$	$^2D_{3/2}$		2	10
^2P	1	0,5	1,5	0,5	4	2					$^2P_{3/2}$	$^2P_{1/2}$	2	6
^2S	0	0,5	0,5	0,5	2	2						$^2S_{1/2}$	1	2
^4D	2	1,5	3,5	0,5	8	2			$^4D_{7/2}$	$^4D_{5/2}$	$^4D_{3/2}$	$^4D_{1/2}$	4	20
^4P	1	1,5	2,5	0,5	6	2				$^4P_{5/2}$	$^4P_{3/2}$	$^4P_{1/2}$	3	12

^4S	0	1,5	1,5	1,5	4	4								$^4S_{3/2}$		1	4
^2D	2	0,5	2,5	1,5	6	4							$^2D_{5/2}$	$^2D_{3/2}$		2	10
^2P	1	0,5	1,5	0,5	4	2								$^2P_{3/2}$	$^2P_{1/2}$	2	6
^2S	0	0,5	0,5	0,5	2	2									$^2S_{1/2}$	1	2
^2K	7	0,5	7,5	6,5	16	14	$^2K_{15/2}$	$^2K_{13/2}$								2	30
^2I	6	0,5	6,5	5,5	14	12		$^2I_{13/2}$	$^2I_{11/2}$							2	26
^2H	5	0,5	5,5	4,5	12	10			$^2H_{11/2}$	$^2H_{9/2}$						2	22
^2H	5	0,5	5,5	4,5	12	10			$^2H_{11/2}$	$^2H_{9/2}$						2	22
^2G	4	0,5	4,5	3,5	10	8				$^2G_{9/2}$	$^2G_{7/2}$					2	18
^2F	3	0,5	3,5	2,5	8	6					$^2F_{7/2}$	$^2F_{5/2}$				2	14
^2H	5	0,5	5,5	4,5	12	10			$^2H_{11/2}$	$^2H_{9/2}$						2	22
^2G	4	0,5	4,5	3,5	10	8				$^2G_{9/2}$	$^2G_{7/2}$					2	18
^2F	3	0,5	3,5	2,5	8	6					$^2F_{7/2}$	$^2F_{5/2}$				2	14
^2G	4	0,5	4,5	3,5	10	8				$^2G_{9/2}$	$^2G_{7/2}$					2	18
^2F	3	0,5	3,5	2,5	8	6					$^2F_{7/2}$	$^2F_{5/2}$				2	14
^2D	2	0,5	2,5	1,5	6	4						$^2D_{5/2}$	$^2D_{3/2}$			2	10
^2F	3	0,5	3,5	2,5	8	6					$^2F_{7/2}$	$^2F_{5/2}$				2	14
^2D	2	0,5	2,5	1,5	6	4						$^2D_{5/2}$	$^2D_{3/2}$			2	10
^2P	1	0,5	1,5	0,5	4	2							$^2P_{3/2}$	$^2P_{1/2}$		2	6
^2F	3	0,5	3,5	2,5	8	6					$^2F_{7/2}$	$^2F_{5/2}$				2	14
^2D	2	0,5	2,5	1,5	6	4						$^2D_{5/2}$	$^2D_{3/2}$			2	10
^2P	1	0,5	1,5	0,5	4	2							$^2P_{3/2}$	$^2P_{1/2}$		2	6
^2P	1	0,5	1,5	0,5	4	2							$^2P_{3/2}$	$^2P_{1/2}$		2	6

^2P	1	0,5	1,5	0,5	4	2							$^2P_{3/2}$	$^2P_{1/2}$	2	6
							2	6	15	26	36	39	35	21	180	1260
