# Supplementary material for: A Theoretical Study of the Occupied and Unoccupied Electronic Structure of High- and Intermediate-Spin Transition Metal Phthalocyaninato (Pc) Complexes: VPc, CrPc, MnPc, and FePc
Source: Nanomaterials (Basel). 2020 Dec 28;11(1):54. doi: 10.3390/nano11010054 (PMC7824030; doi:10.3390/nano11010054)
Supplement: Supplementary file 1 [file nanomaterials-11-00054-s001.zip › Supplementary Material/Table S6.pdf]

Table S6 Symmetries of the $2p^5 3d^5$ system.

	L	S	L+S	|L-S|	J=8	J=7	J=6	J=5	J=4	J=3	J=2	J=1	J=0	States	Deg.
					8	7	6	5	5	3	2	1	0		
^7P	1	3	4	2					$^7P_4$	$^7P_3$	$^7P_2$			3	21
^5P	1	2	3	1						$^5P_3$	$^5P_2$	$^5P_1$		3	15
^5H	5	2	7	3		$^5H_7$	$^5H_6$	$^5H_5$	$^5H_4$	$^5H_3$				5	55
^3H	5	1	6	4			$^3H_6$	$^3H_5$	$^3H_4$					3	33
^5G	4	2	6	2			$^5G_6$	$^5G_5$	$^5G_4$	$^5G_3$	$^5G_2$			5	45
^3G	4	1	5	3				$^3G_5$	$^3G_4$	$^3G_3$				3	27
^5F	3	2	5	1				$^5F_5$	$^5F_4$	$^5F_3$	$^5F_2$	$^5F_1$		5	35
^3F	3	1	4	2					$^3F_4$	$^3F_3$	$^3F_2$			3	21
^5G	4	2	6	2			$^5G_6$	$^5G_5$	$^5G_4$	$^5G_3$	$^5G_2$			5	45
^3G	4	1	5	3				$^3G_5$	$^3G_4$	$^3G_3$				3	27
^5F	3	2	5	1				$^5F_5$	$^5F_4$	$^5F_3$	$^5F_2$	$^5F_1$		5	35
^3F	3	1	4	2					$^3F_4$	$^3F_3$	$^3F_2$			3	21
^5D	2	2	4	0					$^5D_4$	$^5D_3$	$^5D_2$	$^5D_1$	$^5D_0$	5	25
^3D	2	1	3	1						$^3D_3$	$^3D_2$	$^3D_1$		3	15
^5F	3	2	5	1				$^5F_5$	$^5F_4$	$^5F_3$	$^5F_2$	$^5F_1$		5	35
^3F	3	1	4	2					$^3F_4$	$^3F_3$	$^3F_2$			3	21
^5D	2	2	4	0					$^5D_4$	$^5D_3$	$^5D_2$	$^5D_1$	$^5D_0$	5	25
^3D	2	1	3	1						$^3D_3$	$^3D_2$	$^3D_1$		3	15
^5P	1	2	3	1						$^5P_3$	$^5P_2$	$^5P_1$		3	15
^3P	1	1	2	0							$^3P_2$	$^3P_1$	$^3P_0$	3	9
^5D	2	2	4	0					$^5D_4$	$^5D_3$	$^5D_2$	$^5D_1$	$^5D_0$	5	25
^3D	2	1	3	1						$^3D_3$	$^3D_2$	$^3D_1$		3	15
^5P	1	2	3	1						$^5P_3$	$^5P_2$	$^5P_1$		3	15
^3P	1	1	2	0							$^3P_2$	$^3P_1$	$^3P_0$	3	9
^5S	0	2	2	2							$^5S_2$			1	5
^3S	0	1	1	1								$^3S_1$		1	3
^3K	7	1	8	6	$^3K_8$	$^3K_7$	$^3K_6$							3	45
^1K	7	0	7	7		$^1K_7$								1	15

^3I	6	1	7	5	$^3I_7$	$^3I_6$	$^3I_5$					3	39
^1I	6	0	6	6		$^1I_6$						1	13
^3H	5	1	6	4		$^3H_6$	$^3H_5$	$^3H_4$				3	33
^1H	5	0	5	5			$^1H_5$					1	11
^3I	6	1	7	5	$^3I_7$	$^3I_6$	$^3I_5$					3	39
^1I	6	0	6	6		$^1I_6$						1	13
^3H	5	1	6	4		$^3H_6$	$^3H_5$	$^3H_4$				3	33
^1H	5	0	5	5			$^1H_5$					1	11
^3G	4	1	5	3			$^3G_5$	$^3G_4$	$^3G_3$			3	27
^1G	4	0	4	4				$^1G_4$				1	9
^3H	5	1	6	4		$^3H_6$	$^3H_5$	$^3H_4$				3	33
^1H	5	0	5	5			$^1H_5$					1	11
^3G	4	1	5	3			$^3G_5$	$^3G_4$	$^3G_3$			3	27
^1G	4	0	4	4				$^1G_4$				1	9
^3F	3	1	4	2				$^3F_4$	$^3F_3$	$^3F_2$		3	21
^1F	3	0	3	3					$^1F_3$			1	7
^3H	5	1	6	4		$^3H_6$	$^3H_5$	$^3H_4$				3	33
^1H	5	0	5	5			$^1H_5$					1	11
^3G	4	1	5	3			$^3G_5$	$^3G_4$	$^3G_3$			3	27
^1G	4	0	4	4				$^1G_4$				1	9
^3F	3	1	4	2				$^3F_4$	$^3F_3$	$^3F_2$		3	21
^1F	3	0	3	3					$^1F_3$			1	7
^3G	4	1	5	3			$^3G_5$	$^3G_4$	$^3G_3$			3	27
^1G	4	0	4	4				$^1G_4$				1	9
^3F	3	1	4	2				$^3F_4$	$^3F_3$	$^3F_2$		3	21
^1F	3	0	3	3					$^1F_3$			1	7
^3D	2	1	3	1					$^3D_3$	$^3D_2$	$^3D_1$	3	15
^1D	2	0	2	2						$^1D_2$		1	5
^3G	4	1	5	3			$^3G_5$	$^3G_4$	$^3G_3$			3	27
^1G	4	0	4	4				$^1G_4$				1	9

^3F	3	1	4	2	$^3F_4$	$^3F_3$	$^3F_2$			3	21
^1F	3	0	3	3		$^1F_3$				1	7
^3D	2	1	3	1		$^3D_3$	$^3D_2$	$^3D_1$		3	15
^1D	2	0	2	2			$^1D_2$			1	5
^3F	3	1	4	2	$^3F_4$	$^3F_3$	$^3F_2$			3	21
^1F	3	0	3	3		$^1F_3$				1	7
^3D	2	1	3	1		$^3D_3$	$^3D_2$	$^3D_1$		3	15
^1D	2	0	2	2			$^1D_2$			1	5
^3P	1	1	2	0			$^3P_2$	$^3P_1$	$^3P_0$	3	9
^1P	1	0	1	1				$^3P_1$		1	3
^3F	3	1	4	2	$^3F_4$	$^3F_3$	$^3F_2$			3	21
^1F	3	0	3	3		$^1F_3$				1	7
^3D	2	1	3	1		$^3D_3$	$^3D_2$	$^3D_1$		3	15
^1D	2	0	2	2			$^1D_2$			1	5
^3P	1	1	2	0			$^3P_2$	$^3P_1$	$^3P_0$	3	9
^1P	1	0	1	1				$^3P_1$		1	3
^3F	3	1	4	2	$^3F_4$	$^3F_3$	$^3F_2$			3	21
^1F	3	0	3	3		$^1F_3$				1	7
^3D	2	1	3	1		$^3D_3$	$^3D_2$	$^3D_1$		3	15
^1D	2	0	2	2			$^1D_2$			1	5
^3P	1	1	2	0			$^3P_2$	$^3P_1$	$^3P_0$	3	9
^1P	1	0	1	1				$^3P_1$		1	3
^3D	2	1	3	1		$^3D_3$	$^3D_2$	$^3D_1$		3	15
^1D	2	0	2	2			$^1D_2$			1	5
^3P	1	1	2	0			$^3P_2$	$^3P_1$	$^3P_0$	3	9
^1P	1	0	1	1				$^3P_1$		1	3
^3S	0	1	1	1				$^3S_1$		1	3
^1S	0	0	0	0					$^1S_0$	1	1
^3P	1	1	2	0			$^3P_2$	$^3P_1$	$^3P_0$	3	9
^1P	1	0	1	1				$^3P_1$		1	3

1	5	13	24	37	46	45	32	11	214	1512
