# Supplementary material for: A Theoretical Study of the Occupied and Unoccupied Electronic Structure of High- and Intermediate-Spin Transition Metal Phthalocyaninato (Pc) Complexes: VPc, CrPc, MnPc, and FePc
Source: Nanomaterials (Basel). 2020 Dec 28;11(1):54. doi: 10.3390/nano11010054 (PMC7824030; doi:10.3390/nano11010054)
Supplement: Supplementary file 1 [file nanomaterials-11-00054-s001.zip › Supplementary Material/Table S7.pdf]

Table S7 Symmetries of the $2p^5 3d^7$ system.

	L	S	L+S	|L-S|	2(L+S)+1	2(L-S)+1	J=7	J=6	J=5	J=4	J=3	J=2	J=1	J=0	States	Deg.
^5G	4	2,0	6	2	13	5		$^5G_6$	$^5G_5$	$^5G_4$	$^5G_3$	$^5G_2$			5	45
^5F	3	2,0	5	1	11	3			$^5F_5$	$^5F_4$	$^5F_3$	$^5F_2$	$^5F_1$		5	35
^5D	2	2,0	4	0	9	1				$^5D_4$	$^5D_3$	$^5D_2$	$^5D_1$	$^5D_0$	5	25
^3G	4	1,0	5	3	11	7			$^3G_5$	$^3G_4$	$^3G_3$				3	27
^3F	3	1,0	4	2	9	5				$^3F_4$	$^3F_3$	$^3F_2$			3	21
^3D	2	1,0	3	1	7	3					$^3D_3$	$^3D_2$	$^3D_1$		3	15
^5D	2	2,0	4	0	9	1				$^5D_4$	$^5D_3$	$^5D_2$	$^5D_1$	$^5D_0$	5	25
^5P	1	2,0	3	1	7	3					$^5P_3$	$^5P_2$	$^5P_1$		3	15
^5S	0	2,0	2	2	5	5						$^5S_2$			1	5
^3D	2	1,0	3	1	7	3					$^3D_3$	$^3D_2$	$^3D_1$		3	15
^3P	1	1,0	2	0	5	1						$^3P_2$	$^3P_1$	$^3P_0$	3	9
^3S	0	1,0	1	1	3	3							$^3S_1$		1	3
^3I	6	1,0	7	5	15	11	$^3I_7$	$^3I_6$	$^3I_5$						3	39
^3H	5	1,0	6	4	13	9		$^3H_6$	$^3H_5$	$^3H_4$					3	33
^3G	4	1,0	5	3	11	7			$^3G_5$	$^3G_4$	$^3G_3$				3	27
^1I	6	0,0	6	6	13	13		$^1I_6$							1	13
^1H	5	0,0	5	5	11	11			$^1H_5$						1	11
^1G	4	0,0	4	4	9	9				$^1G_4$					1	9
^3H	5	1,0	6	4	13	9		$^3H_6$	$^3H_5$	$^3H_4$					3	33
^3G	4	1,0	5	3	11	7			$^3G_5$	$^3G_4$	$^3G_3$				3	27
^3F	3	1,0	4	2	9	5				$^3F_4$	$^3F_3$	$^3F_2$			3	21

^{1}H	5	0,0	5	5	11	11	$^{1}H_{5}$						1	11
^{1}G	4	0,0	4	4	9	9		$^{1}G_{4}$					1	9
^{1}F	3	0,0	3	3	7	7			$^{1}F_{3}$				1	7
^{3}G	4	1,0	5	3	11	7	$^{3}G_{5}$	$^{3}G_{4}$	$^{3}G_{3}$				3	27
^{3}F	3	1,0	4	2	9	5		$^{3}F_{4}$	$^{3}F_{3}$	$^{3}F_{2}$			3	21
^{3}D	2	1,0	3	1	7	3			$^{3}D_{3}$	$^{3}D_{2}$	$^{3}D_{1}$		3	15
^{1}G	4	0,0	4	4	9	9		$^{1}G_{4}$					1	9
^{1}F	3	0,0	3	3	7	7			$^{1}F_{3}$				1	7
^{1}D	2	0,0	2	2	5	5				$^{1}D_{2}$			1	5
^{3}F	3	1,0	4	2	9	5		$^{3}F_{4}$	$^{3}F_{3}$	$^{3}F_{2}$			3	21
^{3}D	2	1,0	3	1	7	3			$^{3}D_{3}$	$^{3}D_{2}$	$^{3}D_{1}$		3	15
^{3}P	1	1,0	2	0	5	1				$^{3}P_{2}$	$^{3}P_{1}$	$^{3}P_{0}$	3	9
^{1}F	3	0,0	3	3	7	7			$^{1}F_{3}$				1	7
^{1}D	2	0,0	2	2	5	5				$^{1}D_{2}$			1	5
^{1}P	1	0,0	1	1	3	3					$^{3}P_{1}$		1	3
^{3}F	3	1,0	4	2	9	5		$^{3}F_{4}$	$^{3}F_{3}$	$^{3}F_{2}$			3	21
^{3}D	2	1,0	3	1	7	3			$^{3}D_{3}$	$^{3}D_{2}$	$^{3}D_{1}$		3	15
^{3}P	1	1,0	2	0	5	1				$^{3}P_{2}$	$^{3}P_{1}$	$^{3}P_{0}$	3	9
^{1}F	3	0,0	3	3	7	7			$^{1}F_{3}$				1	7
^{1}D	2	0,0	2	2	5	5				$^{1}D_{2}$			1	5
^{1}P	1	0,0	1	1	3	3					$^{3}P_{1}$		1	3
^{3}D	2	1,0	3	1	7	3			$^{3}D_{3}$	$^{3}D_{2}$	$^{3}D_{1}$		3	15
^{3}P	1	1,0	2	0	5	1				$^{3}P_{2}$	$^{3}P_{1}$	$^{3}P_{0}$	3	9

^3S	0	1,0	1	1	3	3							$^3S_1$		1	3
^1D	2	0,0	2	2	5	5						$^1D_2$			1	5
^1P	1	0,0	1	1	3	3							$^1P_1$		1	3
^1S	0	0,0	0	0	1	1								$^1S_0$	1	1
							1	5	11	18	24	25	19	7	110	720
