# Supplementary material for: A Theoretical Study of the Occupied and Unoccupied Electronic Structure of High- and Intermediate-Spin Transition Metal Phthalocyaninato (Pc) Complexes: VPc, CrPc, MnPc, and FePc
Source: Nanomaterials (Basel). 2020 Dec 28;11(1):54. doi: 10.3390/nano11010054 (PMC7824030; doi:10.3390/nano11010054)
Supplement: Supplementary file 1 [file nanomaterials-11-00054-s001.zip › Supplementary Material/Tables S1 ΓÇô S4.docx]

A theoretical study of the occupied and unoccupied electronic structure of high- and intermediate-spin transition metal phthalocyaninato (Pc) complexes: VPc, CrPc, MnPc, and FePc.

Silvia Carlotto,*^a^ Mauro Sambi, ^a^ Francesco Sedona, ^a^ Andrea Vittadini^b^ and Maurizio Casarin*^a,b^

Supplementary Material

Table S1. Optimized BP86 Cartesian coordinates for HS VPc.

V 0.000000 0.000000 0.000000

N 0.000000 1.995751 0.000000

N 1.995751 0.000000 0.000000

N 0.000000 -1.995751 0.000000

N -1.995751 0.000000 0.000000

N 2.407785 2.407785 0.000000

N 2.407785 -2.407785 0.000000

N -2.407785 2.407785 0.000000

N -2.407785 -2.407785 0.000000

C 2.801815 1.134611 0.000000

C 2.801815 -1.134611 0.000000

C 1.134611 -2.801815 0.000000

C -1.134611 -2.801815 0.000000

C -2.801815 -1.134611 0.000000

C -2.801815 1.134611 0.000000

C -1.134611 2.801815 0.000000

C 1.134611 2.801815 0.000000

C 4.193548 0.708990 0.000000

C 4.193548 -0.708990 0.000000

C 0.708990 -4.193548 0.000000

C -0.708990 -4.193548 0.000000

C -4.193548 -0.708990 0.000000

C -4.193548 0.708990 0.000000

C -0.708990 4.193548 0.000000

C 0.708990 4.193548 0.000000

C 5.393589 1.427772 0.000000

C 5.393589 -1.427772 0.000000

C 1.427772 -5.393589 0.000000

C -1.427772 -5.393589 0.000000

C -5.393589 -1.427772 0.000000

C -5.393589 1.427772 0.000000

C -1.427772 5.393589 0.000000

C 1.427772 5.393589 0.000000

C 6.586928 0.705255 0.000000

C 6.586928 -0.705255 0.000000

C 0.705255 -6.586928 0.000000

C -0.705255 -6.586928 0.000000

C -6.586928 -0.705255 0.000000

C -6.586928 0.705255 0.000000

C -0.705255 6.586928 0.000000

C 0.705255 6.586928 0.000000

H 5.386110 2.518079 0.000000

H 5.386110 -2.518079 0.000000

H 2.518079 -5.386110 0.000000

H -2.518079 -5.386110 0.000000

H -5.386110 -2.518079 0.000000

H -5.386110 2.518079 0.000000

H -2.518079 5.386110 0.000000

H 2.518079 5.386110 0.000000

H -1.237164 -7.539685 0.000000

H 1.237164 -7.539685 0.000000

H 7.539685 -1.237164 0.000000

H 7.539685 1.237164 0.000000

H 1.237164 7.539685 0.000000

H -1.237164 7.539685 0.000000

H -7.539685 1.237164 0.000000

H -7.539685 -1.237164 0.000000

Table S2. Optimized BP86 Cartesian coordinates for HS CrPc.

Cr 0.000000 0.000000 0.000000

N 0.000000 1.982180 0.000000

N 1.982180 0.000000 0.000000

N 0.000000 -1.982180 0.000000

N -1.982180 0.000000 0.000000

N 2.400852 2.400852 0.000000

N 2.400852 -2.400852 0.000000

N -2.400852 2.400852 0.000000

N -2.400852 -2.400852 0.000000

C 2.789117 1.128550 0.000000

C 2.789117 -1.128550 0.000000

C 1.128550 -2.789117 0.000000

C -1.128550 -2.789117 0.000000

C -2.789117 -1.128550 0.000000

C -2.789117 1.128550 0.000000

C -1.128550 2.789117 0.000000

C 1.128550 2.789117 0.000000

C 4.184723 0.706646 0.000000

C 4.184723 -0.706646 0.000000

C 0.706646 -4.184723 0.000000

C -0.706646 -4.184723 0.000000

C -4.184723 -0.706646 0.000000

C -4.184723 0.706646 0.000000

C -0.706646 4.184723 0.000000

C 0.706646 4.184723 0.000000

C 5.382239 1.427343 0.000000

C 5.382239 -1.427343 0.000000

C 1.427343 -5.382239 0.000000

C -1.427343 -5.382239 0.000000

C -5.382239 -1.427343 0.000000

C -5.382239 1.427343 0.000000

C -1.427343 5.382239 0.000000

C 1.427343 5.382239 0.000000

C 6.576695 0.704571 0.000000

C 6.576695 -0.704571 0.000000

C 0.704571 -6.576695 0.000000

C -0.704571 -6.576695 0.000000

C -6.576695 -0.704571 0.000000

C -6.576695 0.704571 0.000000

C -0.704571 6.576695 0.000000

C 0.704571 6.576695 0.000000

H 5.374210 2.517645 0.000000

H 5.374210 -2.517645 0.000000

H 2.517645 -5.374210 0.000000

H -2.517645 -5.374210 0.000000

H -5.374210 -2.517645 0.000000

H -5.374210 2.517645 0.000000

H -2.517645 5.374210 0.000000

H 2.517645 5.374210 0.000000

H -1.236934 -7.529167 0.000000

H 1.236934 -7.529167 0.000000

H 7.529167 -1.236934 0.000000

H 7.529167 1.236934 0.000000

H 1.236934 7.529167 0.000000

H -1.236934 7.529167 0.000000

H -7.529167 1.236934 0.000000

H -7.529167 -1.236934 0.000000

Table S3. Optimized BP86 Cartesian coordinates for IS MnPc.

Mn 0.000000 0.000000 0.000000

N 0.000000 1.951773 0.000000

N 1.951773 0.000000 0.000000

N 0.000000 -1.951773 0.000000

N -1.951773 0.000000 0.000000

N 2.396997 2.396997 0.000000

N 2.396997 -2.396997 0.000000

N -2.396997 2.396997 0.000000

N -2.396997 -2.396997 0.000000

C 2.773696 1.127805 0.000000

C 2.773696 -1.127805 0.000000

C 1.127805 -2.773696 0.000000

C -1.127805 -2.773696 0.000000

C -2.773696 -1.127805 0.000000

C -2.773696 1.127805 0.000000

C -1.127805 2.773696 0.000000

C 1.127805 2.773696 0.000000

C 4.163375 0.705262 0.000000

C 4.163375 -0.705262 0.000000

C 0.705262 -4.163375 0.000000

C -0.705262 -4.163375 0.000000

C -4.163375 -0.705262 0.000000

C -4.163375 0.705262 0.000000

C -0.705262 4.163375 0.000000

C 0.705262 4.163375 0.000000

C 5.361079 1.427873 0.000000

C 5.361079 -1.427873 0.000000

C 1.427873 -5.361079 0.000000

C -1.427873 -5.361079 0.000000

C -5.361079 -1.427873 0.000000

C -5.361079 1.427873 0.000000

C -1.427873 5.361079 0.000000

C 1.427873 5.361079 0.000000

C 6.554490 0.705235 0.000000

C 6.554490 -0.705235 0.000000

C 0.705235 -6.554490 0.000000

C -0.705235 -6.554490 0.000000

C -6.554490 -0.705235 0.000000

C -6.554490 0.705235 0.000000

C -0.705235 6.554490 0.000000

C 0.705235 6.554490 0.000000

H 5.352581 2.518106 0.000000

H 5.352581 -2.518106 0.000000

H 2.518106 -5.352581 0.000000

H -2.518106 -5.352581 0.000000

H -5.352581 -2.518106 0.000000

H -5.352581 2.518106 0.000000

H -2.518106 5.352581 0.000000

H 2.518106 5.352581 0.000000

H -1.237543 -7.506985 0.000000

H 1.237543 -7.506985 0.000000

H 7.506985 -1.237543 0.000000

H 7.506985 1.237543 0.000000

H 1.237543 7.506985 0.000000

H -1.237543 7.506985 0.000000

H -7.506985 1.237543 0.000000

H -7.506985 -1.237543 0.000000

Table S4. Optimized BP86 Cartesian coordinates for IS FePc.

Fe 0.000000 0.000000 0.000000

N 0.000000 1.938380 0.000000

N 1.938380 0.000000 0.000000

N 0.000000 -1.938380 0.000000

N -1.938380 0.000000 0.000000

N 2.392934 2.392934 0.000000

N 2.392934 -2.392934 0.000000

N -2.392934 2.392934 0.000000

N -2.392934 -2.392934 0.000000

C 2.758900 1.118667 0.000000

C 2.758900 -1.118667 0.000000

C 1.118667 -2.758900 0.000000

C -1.118667 -2.758900 0.000000

C -2.758900 -1.118667 0.000000

C -2.758900 1.118667 0.000000

C -1.118667 2.758900 0.000000

C 1.118667 2.758900 0.000000

C 4.153278 0.704499 0.000000

C 4.153278 -0.704499 0.000000

C 0.704499 -4.153278 0.000000

C -0.704499 -4.153278 0.000000

C -4.153278 -0.704499 0.000000

C -4.153278 0.704499 0.000000

C -0.704499 4.153278 0.000000

C 0.704499 4.153278 0.000000

C 5.349405 1.428580 0.000000

C 5.349405 -1.428580 0.000000

C 1.428580 -5.349405 0.000000

C -1.428580 -5.349405 0.000000

C -5.349405 -1.428580 0.000000

C -5.349405 1.428580 0.000000

C -1.428580 5.349405 0.000000

C 1.428580 5.349405 0.000000

C 6.542603 0.705244 0.000000

C 6.542603 -0.705244 0.000000

C 0.705244 -6.542603 0.000000

C -0.705244 -6.542603 0.000000

C -6.542603 -0.705244 0.000000

C -6.542603 0.705244 0.000000

C -0.705244 6.542603 0.000000

C 0.705244 6.542603 0.000000

H 5.340426 2.518759 0.000000

H 5.340426 -2.518759 0.000000

H 2.518759 -5.340426 0.000000

H -2.518759 -5.340426 0.000000

H -5.340426 -2.518759 0.000000

H -5.340426 2.518759 0.000000

H -2.518759 5.340426 0.000000

H 2.518759 5.340426 0.000000

H -1.236959 -7.495414 0.000000

H 1.236959 -7.495414 0.000000

H 7.495414 -1.236959 0.000000

H 7.495414 1.236959 0.000000

H 1.236959 7.495414 0.000000

H -1.236959 7.495414 0.000000

H -7.495414 1.236959 0.000000

H -7.495414 -1.236959 0.000000
